# A Case of Dysentery

**Published:** 1887-07

**Authors:** Clarence Willard Butler


### A CASE OF DYSENTERY.

CLARENCE WILLARD BUTLER, M. D.

"The shot of accident; the dart of chance."—*Othello.*

At about seven A. M. one warm September morning in 1885 I was called to see Mrs. B., a tall, dark-complexioned woman of middle age and of an exceedingly nervous temperament. Unable to go to her at once I sent one dose of Nux vom. high. At half-past eight A. M. I was called again and hastened to her bedside. The Nux had not relieved her in the least, and she now presented the following symptoms: Sudden severe urging to stool and as often as every ten minutes. Painful tenesmus with stool, which was relieved at once and entirely by the evacuation of the rectum. The stool itself consisted of about a tablespoonful of transparent mucus, in appearance exactly like the white of egg, but perhaps a little more jelly-like in consistency.

Patient apprehensive of severe illness, exceedingly nervous, even to tremulousness, with sadness.

Not knowing what to give, I took the coachman to my office to get medicine, thinking that I would study up the case there. Arriving at the office, I found it filled with waiting patients, all of whom were provoked to find me away during office hours, and all of whom were in a hurry. So I hastily consulted Allen's *Symptom Register* and found "Stool like white of egg," Carb., Crot.-tig., Dios., K. Iod.

Of these *K. Iod.* alone had the apprehensiveness "of misfortune" (the nearest I could come to her mental condition), and being forced by circumstances into making what sportsmen call a "pop shot," sent her one dose of this remedy in the 200th potency, B. & T., which she got about nine o'clock. Two hours later I hastened to see her again and found her much better. After taking the Kali iod. she had a stool almost immediately unchanged in character. At ten A. M. another stool, consisting of the same transparent mucus and some fecal matter, with a decided modification of all the accompanying symptoms. She had one more stool toward evening, fecal and pasty, and with no mucus at all in it.

The next day she had no stool and the third day a natural fecal evacuation at her usual time in the morning. As all her nervousness had disappeared with the subsidence of the dysenteric symptoms, she needed no further medicine and received none.

Since Mrs. B. had already received one dose of medicine similar in appearance to the dose of Kali iod., without relief, since she was sure that she would be seriously ill, and since I made the prescription without faith, based, as it was, upon a hasty and insufficient diagnosis for the remedy, this prompt relief cannot be claimed for the faith, mind, or prayer cure. (Indeed, such prayers as I was moved to offer, were, I fear, somewhat mixed.) Again, while symptoms 5, 8, 17, 422, and 427 in Allen's *Encyclopædia* do not present such a clear picture of the case as is always desirable (though often impossible) in prescribing, still they do show such a decided relation to the case as to convince any but the chronically skeptical that Kali was the curative agent, and since the symptoms were clearly cut, the condition peculiar and severe, and the relief prompt, this has seemed a case worth reporting and—remembering.

